# Untargeted metabolomics of purple and orange-fleshed sweet potatoes reveals a large structural diversity of anthocyanins and flavonoids

**DOI:** 10.1038/s41598-021-95901-y

**Published:** 2021-08-12

**Authors:** Alexandra A. Bennett, Elizabeth H. Mahood, Kai Fan, Gaurav D. Moghe

**Affiliations:** 1 Plant Biology Section, School of Integrative Plant Science, Cornell University, Ithaca, USA; 2 Key Laboratory of Ministry of Education for Genetics, Breeding and Multiple Utilization of Crops, College of Agriculture, Fujian Agriculture and Forestry University, Fuzhou, 350002 People’s Republic of China; 3 Present Address: Institute of Analytical Chemistry, Department of Chemistry, Universität Für Bodenkultur Wien, 1190 Vienna, Austria

**Keywords:** Metabolomics, Mass spectrometry, Secondary metabolism

## Abstract

Anthocyanins are economically valuable phytochemicals of significant relevance to human health. Industrially extracted from multiple fruit and vegetable sources, anthocyanin yield and profiles can vary between sources and growing conditions. In this study, we focused on three purple-fleshed and one orange-fleshed cultivars of sweet potato—a warm-weather, nutritious crop of substantial interest to growers in northern, cooler latitudes—to determine the yield and diversity of anthocyanins and flavonoids. Acidified ethanol extraction of lyophilized roots yielded ~ 800 mg average anthocyanins/100 g dry weight from all three cultivars. UHPLC-DAD-Orbitrap analysis of sweet potato extracts identified 18 high-confidence, mostly acylated peonidin and cyanidin derivatives contributing to > 90% of the total anthocyanin signal. Further assessment of the untargeted Liquid Chromatography–Tandem Mass Spectrometry data using deep learning and molecular networking identified over 350 flavonoid peaks with variable distributions in different sweet potato cultivars. These results provide a novel insight into anthocyanin content of purple-fleshed sweet potatoes grown in the northern latitudes, and reveal the large structural diversity of anthocyanins and flavonoids in this popular crop.

## Introduction

Anthocyanins are water-soluble phytochemical pigments of significant health and economic value, which belong to a class of polyphenolic compounds called flavonoids. Found in many fruits and vegetables, flavonoids possess antioxidant activities of benefit in managing ageing, stress, cancer and other health conditions, which makes them desirable for cosmetic, nutritional and health industry applications^1–3^. Because of their color properties, anthocyanins are also of significant interest as natural food coloring agents^3,4^. Due to these diverse uses, the global market for flavonoids is expected to exceed $1 billion by 2026^5^.

Flavonoids are downstream products of the phenylpropanoid pathway and are defined by presence of a flavone ring structure comprised of three rings termed A, B and C^6^. Anthocyanidins—the aglycone of anthocyanins—are differentiated from other flavonoids by the presence of a positively charged oxygen on the C ring which results in a flavylium cation. Modification through glycosylation, acylation, methylation, and hydroxylation reactions can stabilize these compounds, making them more resistant to changes in pH, temperature and ultraviolet light, and thus, more useful for commercial applications^4,6^. The most common modification is glycosylation, producing anthocyanins. Currently, the major sources of industrial production of anthocyanins include skins of grapes processed in the wine industry, berries, black carrots, red cabbage, and purple-flesh sweet potatoes^7,8^.

Sweet potato (*Ipomoea batatas*) is an attractive crop for anthocyanin extraction. While the major cultivated varieties are orange-fleshed and rich in carotenoids such as β-carotene^9^, there are also purple-fleshed varieties. One meta-analysis compared anthocyanin yields from various sources and estimated 84–174 mg anthocyanins/100 g fresh weight from purple-flesh sweet potatoes compared to 6–600 mg/100 g fresh weight from grapes and ~ 25 mg/100 g fresh weight from red cabbage^10^. However, unlike fruits, sweet potato roots produce high biomass, store better, and can be cultivated on large scale. In addition, they primarily make more stable, acylated anthocyanins^10,11^, unlike many non-acylated fruit anthocyanins^4,12^. At least 27 anthocyanins are known to be present in different sweet potato cultivars^11,13–15^, in addition to other health promoting phenolic compounds^13^. Studies in sweet potato have revealed primarily peonidin and cyanidin derived anthocyanins^14,16^, but the overall extent of anthocyanin diversity is not known.

In the United States, sweet potato is a crop primarily grown in warmer states with long growing seasons, such as North Carolina—which leads the country in sweet potato production^17^—Louisiana, Mississippi, and California. These states accounted for > 90% of the 3.1 billion pounds of sweet potato grown in 2015 in the USA^17^. Originating from tropical areas in Central America and northwestern South America^18^, sweet potato is adapted to warmer climates and soils and a longer growing season. However, many regions in the northern latitudes in the US and Canada are increasingly interested in cultivation of this crop due to its economic value in grocery markets as well as for industrial products^19–22^. Geneva, New York, where this project was conducted has, on an average, ~ 5 °C lower daytime temperatures and 5–12 °C lower nighttime temperatures in the US’s primary growing months of June–September, than principal sweet potato growing regions such as Raleigh (USA), Xuzhou (China), Lagos (Nigeria) as well as Okinawa (Japan) where the popular purple-fleshed ‘Okinawan’ sweet potato originated (Supplementary Fig. 1). This temperature gap and the shorter growing season are representative of conditions across the northern latitudes, thus necessitating the evaluation of sweet potato growth and anthocyanin yield in cooler climates for a better understanding of their economic potential. We note that the goal of this study was not to compare whether individual varieties produce higher/lower anthocyanin yields in cooler climates vs. warmer climates, but only to determine the levels and types of anthocyanins produced in cooler climates.

Thus, we focused on the anthocyanin yield and content of three purple-fleshed varieties grown in upstate New York. One advantage of growing sweet potatoes in the north is a relative absence of natural pests, which leads to less pesticide application and a resulting organic cultivation. Furthermore, cold stress is known to induce anthocyanin production in other species^23–25^. While high yield orange-fleshed varieties such as Beauregard and Covington have been studied in more detail for their growth characteristics^26–28^, the anthocyanin content of purple-fleshed varieties grown in the north has not been assessed. In addition, a comprehensive computational evaluation of flavonoid content in sweet potatoes, especially purple ones, has not been made before. New tools that employ deep learning and allow high-throughput computational evaluation of Liquid Chromatography–Tandem Mass Spectrometry (LC–MS/MS) data can provide novel insights into anthocyanin and flavonoid diversity. Therefore, in this study, we first evaluated the biomass yield of three easily available purple-fleshed sweet potato cultivars and compared it to the most popular orange-fleshed variety Beauregard. Then, we selected the most optimal low temperature anthocyanin extraction method and evaluated monomeric anthocyanin content. Finally, we used machine learning and molecular networking to evaluate flavonoid diversity in the three purple-fleshed cultivars. Our results suggest substantial diversity and yield of these compounds, and reinforce the value of purple sweet potatoes as industrial sources of anthocyanins and flavonoids.

## Materials and methods

### Growing conditions

Slips of four sweet potato cultivars were obtained as follows: ‘Kotobuki’ and ‘Purple Passion’ from George’s Plant Farm^29,30^, and ‘All Purple Sweet Potato’ and ‘Beauregard’ from Southern Exposure Seed Exchange. These varieties were chosen for their ease of availability and noted dark purple skin and purple flesh color, conducive for high anthocyanin yields. ‘Kotobuki’ is sometimes described as Japanese Sweet Potato with a red skin and white flesh, but the ‘Kotobuki’ we refer to here is a purple-fleshed, purple-skinned variety obtained from the above source. Slips were transplanted to “Cornell Mix” soil substrate^31^ and maintained within a greenhouse at Cornell University until field season. Slips were transplanted to a field in Geneva, NY onto raised plastic beds with 1.8 m centers and 45 cm spacing. Plants were maintained with drip irrigation and fertigation until harvest after 106 days. Sweet potato roots were cured for 3 weeks at room temperature, washed and were kept at room temperature (~ 18 °C) for another 6 weeks at ambient humidity. Sweet potatoes were finally moved to long term storage at 10 °C and ~ 80% relative humidity. The weight, length, and circumference were measured 1 month after harvest for each sweet potato.

### Solvents and chemicals

The following ACS grade reagents were sourced from VWR, Radnor, Pennsylvania, USA: 95% ethanol, ≥ 88% formic acid and ≥ 99.7% glacial acetic acid, 36.5–38% hydrochloric acid, potassium chloride and ≥ 99% sodium acetate. The standard ≥ 90% cyanin chloride was purchased from Santa Cruz Biotechnology, Dallas, Texas, USA. Ultra-high purity water was generated by an Elga PureLab Ultra reverse osmosis system equipped with a LC182 purification cartridge. LC–MS grade acetonitrile, water, and formic acid were obtained from ThermoFisher Scientific (Waltham, Massachusetts, USA).

### Comparisons of processing methods for anthocyanin extraction

Five randomized replicates from the sweet potato cultivar ‘All Purple Sweet Potato’ were sliced into 5 mm thick discs and mixed. Each replicate was divided into three portions of ~ 100 g each for three different processing methods (raw, frozen and lyophilized). The raw portion was ground in a grain mill and extracted. The second and third portions were snap frozen in liquid nitrogen. The second portion was then ground with the aid of dry ice and given 2 days to vent at  -80 °C before extraction. The third portion was lyophilized over a 24-h period and weighed before grinding. 50 g of each method wase weighed out (with lyophilized adjusted to reflect fresh weight). Samples were extracted in 50 mL of 75% ethanol with 10% acetic acid. Anthocyanin extraction was performed in the dark following a standard protocol^32^. Homogenized tissue was extracted in designated solvent for 60 min on an VWR^®^ Variable Speed Rocker set to the highest speed. Liquid was vacuum filtered with a Buchner funnel lined with Whatman’s student grade filter paper and stored at  -20 °C until anthocyanins were quantified with a spectrophotometer. For cultivar comparisons, four replicates of the three purple-fleshed cultivars under study were processed via lyophilization method as described above, with the exception that 20 g dry weight was extracted in 50 mL.

### Quantification of anthocyanins

Cyanin chloride (referred to here as cyanin) was dissolved in methanol containing 1% formic acid to a concentration of 8 mM. 40 μL of cyanin solution was then mixed with 1960 μL of 25 mM potassium chloride pH 1.0, and another 40 μL cyanin solution was mixed with 1960 μL of 0.4 M sodium acetate pH 4.5. The stocks were given 15 min to reach equilibrium, and then diluted two-fold serially to generate 7 concentrations ranging from 160 to 1.25 μM (this reflects a sample concentration of 8–62.5 μM). Cyanin solutions were then measured in triplicate with a Varian Cary 50 Bio UV–Visible spectrophotometer within 1 h and analyzed via pH differential method^33^. The computer system monitored and analyzed data using Varian Cary WinUV Simple Reads software version 4.10 (build 464). The same protocol was used for measuring anthocyanins from root extracts. Depending on the pH, potassium chloride or sodium acetate was used as blanks. Equilibrated sample and standard curve absorbance measurements were then taken at 520 nm and 700 nm by the spectrophotometer^33^. Final absorbance values were calculated through the pH differential method as follows:

$$A = (A_{520} {-} A_{700} )_{pH\;1.0} {-} (A_{520} {-} A_{700} )_{pH\; 4.5}$$

The Kolmogorov–Smirnov test was performed in Statistical Analysis System’s (SAS) JMP Pro software v14.3.0. Cyanin values were used to generate a standard curve. The linear regression from this standard curve was used to calculate mg/g concentrations of each condition and cultivar in Microsoft Excel 365. Corrections were performed for both dry weight and fresh weight measurements. A recent study^34^ suggested some issues with monomeric anthocyanin estimates derived from the pH differential method (see “Discussion”). While these were not taken into consideration in the present study, our and other previous estimates may need to be upwardly revised to account for anthocyanins missed in the pH differential method.

### Estimation of concentrations and dilution corrections

For processing comparisons, samples were reported in dry weight equivalence. To make the experiment comparable across all three processing conditions, dilution from water contained within fresh roots needed to be accounted for. This was done through dilution equations:

$$c_{1} \cdot V_{1} = c_{2} \cdot V_{2}$$

In this equation, *c*_1_ is the original concentration (in mM from the standard curve) of the lyophilized extract measured and *V*_1_ is the 50 mL of solvent used for extraction plus the average water within the root. *c*_2_ reflects what the concentration of the sample would be if this were a dry weight extraction and there were only 50 mL of solvent. Thus, *V*_2_ is 50 mL. This adjusted mM value was then used to calculate dry weight mg/g values for non-lyophilized samples. Similar adjustments (Supplementary File 2) were made to calculate fresh weight anthocyanin yields for lyophilized samples.

### Mass spectrometric analysis

One mL of anthocyanin extract was transferred to amber High Performance Liquid Chromatography (HPLC) vials (VWR 46610-726). Samples were separated with a Dionex UltiMate 3000 Ultra HPLC (UHPLC) system and Phenomenex Kinetex F5 column (00D-4722-AN, 1.7 µm particle size, 100 Å pore size, 100 mm length, 2.10 mm Internal Diameter) at a flow rate of 0.6 mL/min. Solvent A was Ultrapure H_2_O and Solvent B was acetonitrile, both with 3% formic acid. Solvent gradient was as follows (values in Time [min]: %B): 0.0: 5%, 1.0: 12%, 7.5: 15%, 8.0: 40%, 9.0: 14%, 9.0: 5%, 10.0: 5%. After separation, anthocyanins were detected by a Dionex UltiMate 3000 diode array and multiple-wavelength detector (DAD) at 520 nm with a 700 nm reference in addition to UV–VIS full spectrum. Mass spectrometry was performed in positive mode, with a MS1 resolution of 70,000 and scan range of 200–20,000 *m/z* in profile mode. A Thermo Fisher Orbitrap Q-Exactive detected anthocyanins through data dependent MS2 (DDMS2) scans after the DAD. 520 nm is a midrange maximum absorbance (λ_max_) value amongst anthocyanins while 700 nm is used to correct for haze as there is no absorbance by anthocyanins at this wavelength. DDMS2 was performed for top ten MS1 ions, with AGC target of 5e5 and resolution of 17,500.

Raw data was converted to Analysis Based File (ABF) format using Reifycs ABF Converter and imported into MS-DIALv4.24^35^. Data from samples was filtered and aligned using MS-DIAL and data on all aligned metabolites was exported in mascot generic format (MGF). Anthocyanins were selected manually in MS-DIAL based upon literature review^36–38^ and high abundance aglycone signature fragments (*m/z*: 287.06 for cyanidin, 271.06 for pelargonidin, 303.05 for delphinidin, 301.07 for peonidin and 317.07 for petunidin) and exported to MS-Finder v3.44 ^40^ to obtain their chemical formulas. ThermoFisher Chromeleon was used to manually align 520 nm peak data from the DAD to a subset of high intensity MS anthocyanin peaks previously identified. Peak intensities from UV–VIS data was used to calculate percent anthocyanin composition in Chromeleon.

### Identification of flavonoids using fragment-based and deep learning-based approaches

All metabolites exported in Mascot Generic Format from MS-DIAL^35^ were filtered using a custom Python script (https://github.com/lizmahood/flavonoid_processing) to identify anthocyanin-like peaks (including flavonoids of similar masses) across all cultivar LC–MS/MS datasets. This script selected all peaks containing both the anthocyanidin and its mono or disaccharide derivative as fragments with intensities > 3000. For anthocyanidins, we used the nominal *m/z* values for pelargonidin (271), cyanidin (287), peonidin (301), petunidin (317), delphinidin (303), malvidin (331), rosinidin (315), and capensinidin (345). Monosaccharides considered included all masses for hexose (162), deoxyhexose (146) and pentose (132) sugars, while the six disaccharides were pairwise combinations of the monosaccharide masses.

Spectra from all metabolites were also submitted to CANOPUS (included in SIRIUS v4.5.2^39^) for structural classification. All parameters were kept at default values except: “Instrument” was set to Orbitrap, and “Candidates” was set to 3. Each compound’s posterior probability score associated with flavonoid prediction was used to assess the quality of each compound’s classification. Compounds with scores > 0.63337 for the Flavonoid class—the minimum posterior probability score associated with correct class prediction achieved by any of the 18 anthocyanins—were considered in downstream analyses.

### Molecular networking analysis

A new Mascot Generic Format file was created containing entries for the 18 manually identified anthocyanins as well as for any flavonoid identified by either of the two above methods. Before network construction, any fragment peak with abundance < 3000 was removed. MS-Finder v3.44^40^ was used to generate a molecular network (MS similarity cutoff of 70%) and export the node and edge files. Seventeen peaks with after decimal *m/z* values between 0.40–0.80—atypical of flavonoids and anthocyanins—were found in the network. Sixteen of these were identified in-house, and therefore kept in the final network due to presence of appropriate MS/MS aglycone and glycone masses. The one predicted by CANOPUS alone was not retained as it contained a peonidin core fragment, but no glycone masses. Nodes and edges were imported into Cytoscape v3.8.0^41^ and displayed using Prefuse Force Directed Layout for figure generation. The heatmap shown in Figure 4 was created in R v4.0.4 using the pheatmap package.

### Conformance to guidelines for experimental work

All field and lab experiments were conducted in accordance with experimental guidelines set by Cornell University Greenhouses and Occupational and Environmental Health and Safety (e.g. Worker Protection Standards training). Necessary permissions were obtained from the Horticulture and Plant Biology section chairs for field planting. Experimental work in the lab is approved by the Cornell Institutional Biosafety Review Board.

## Results

### Purple-fleshed sweet potatoes produce lower per-root yield than orange-fleshed sweet potatoes

**Figure 1:**
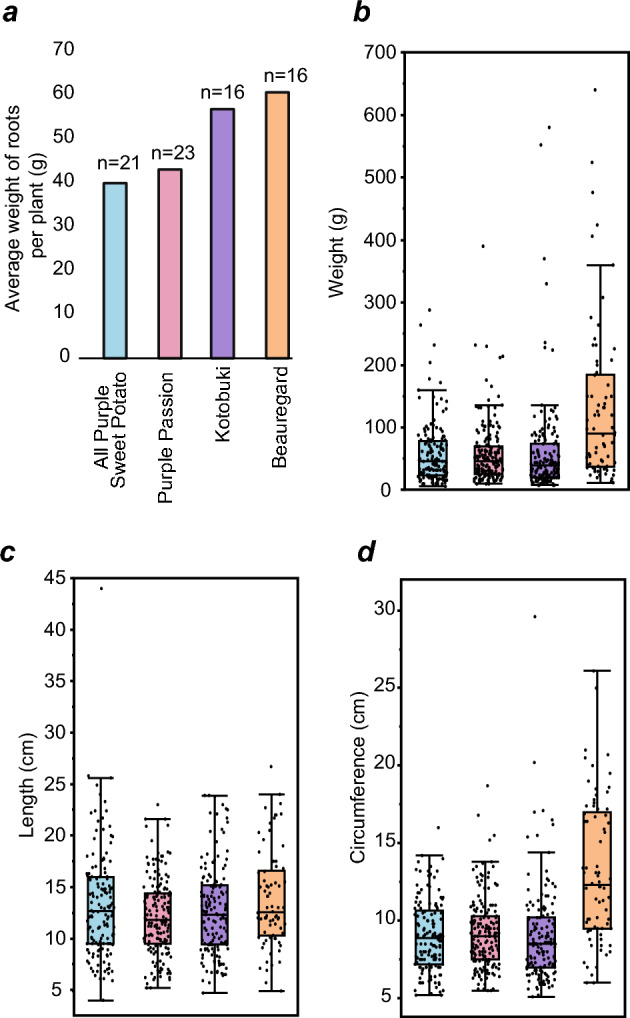
Phenotyping of studied sweet potato cultivars. (**a**) Average weight (in grams) of roots per plant, obtained by dividing total weight of all sweet potatoes for a given cultivar by the number of plants harvested. Number of plants for each cultivar is indicated above the bar graph. (**b**–**d**) Weight, length and circumference distributions for each sweet potato variety. Colors used for each cultivar are the same across all panels.

Three purple-fleshed and one orange-fleshed sweet potato varieties were grown from slips in Geneva, NY for 106 days, and were phenotyped after harvesting (Fig. 1; Supplementary File 1). The orange-fleshed variety ‘Beauregard’ is one of the most popular sweet potato varieties in the US, and hence was grown for yield comparisons. Yield metrics for ‘All Purple Sweet Potato’ and ‘Purple Passion’ were not significantly different (Kolmogorov–Smirnov [KS] test, p = 0.84), however, ‘Kotobuki’ performed better than these two varieties (Fig. 1a). While it had a similar average root weight, circumference, and length, the average number of sweet potatoes per plant was 9.4—compared to 7.3 for the other two—resulting in greater yield per plant. The orange-fleshed cultivar ‘Beauregard’ outperformed all three purple varieties in biomass (KS test, p < 1.2e-5 for all comparisons). The average Beauregard plant produced sweet potatoes that were almost 2X as heavy and 1.5X as wide as ‘Kotobuki’, despite having the lowest average number of sweet potatoes harvested per plant (4.6). All three purple cultivars were studied to determine their anthocyanin yield and profiles.

### Lyophilization produces consistent yield among the different cold extraction methods

Anthocyanins are relatively hydrophobic and have poor extraction abilities in a neutral pH aqueous solution. Thus, solvents such as acidified ethanol, acidified methanol, and acetone:chloroform, are typically used in the extraction of anthocyanins^10^. Given material cost and toxicity are important considerations for future food-related industrial applications, we only used acidified 75% ethanol for extractions. Heat-based processing methods such as boiling, blanching and pressure cooking are sometimes studied to determine how cooking practices change anthocyanin levels. Since the main goal of this study was to determine anthocyanin content, we primarily focused on assessing cold-associated methods used in industrial processing, storage and transport, namely homogenization of raw tissue, snap freezing, and lyophilization, using the ‘All Purple Sweet Potato’ variety as an exemplar of purple-fleshed sweet potatoes.

**Figure 2:**
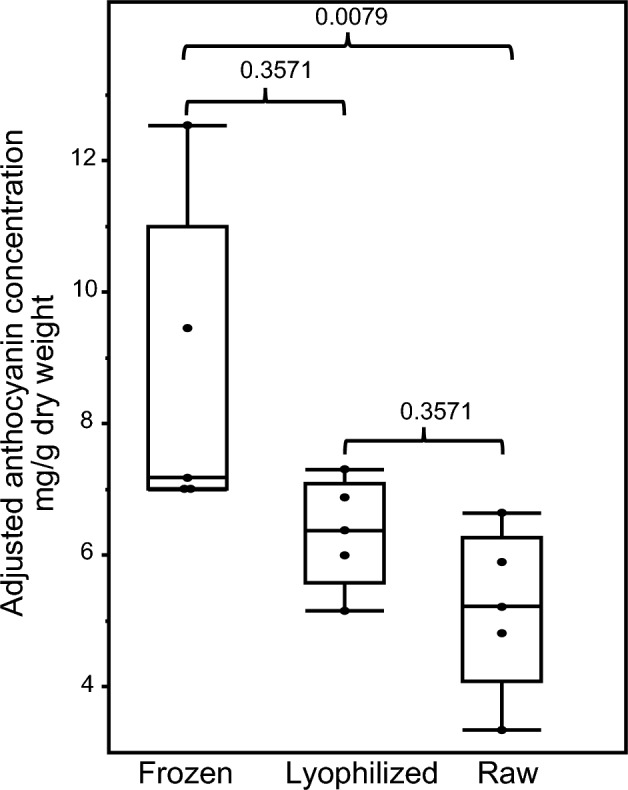
Quantification of anthocyanins obtained using different processing methods using the representative anthocyanin cyanidin 3,5-diglucoside (cyanin) as standard. Total monomeric anthocyanin yields (cyanin equivalence) of purple fleshed sweet potatoes were compared among three extraction and processing strategies, namely freezing, lyophilizing, and raw, using ‘All Purple Sweet Potato.’ Adjustments performed for dry weight are noted in the “Methods” section and shown in Supplementary File 2. Non-parametric Kolmogorov–Smirnov test was utilized to estimate p-values.

Freezing samples in liquid nitrogen with or without subsequent lyophilization resulted in the highest levels of anthocyanins extracted—6.4 and 7.2 median mg/g dry weight, respectively (Fig. 2; Supplementary File 2). Although raw frozen had slightly higher (but not statistically significantly different) yield than lyophilized, the standard deviation for freezing without lyophilization (2.4 mg/g) was ~ 3X freezing with subsequent lyophilization (0.8 mg/g). This suggested that lyophilization resulted in more reproducible and high anthocyanin yields, possibly because of the variable water content in samples without lyophilization. Thus, sweet potatoes for further experiments were snap frozen and lyophilized before extraction in 75% acidified ethanol. After standardizing this optimal anthocyanin processing method in one variety, we used it to assess the anthocyanin variability among different sweet potato cultivars.

### Eighteen high-confidence anthocyanins were identified across all three purple sweet potatoes

**Figure 3:**
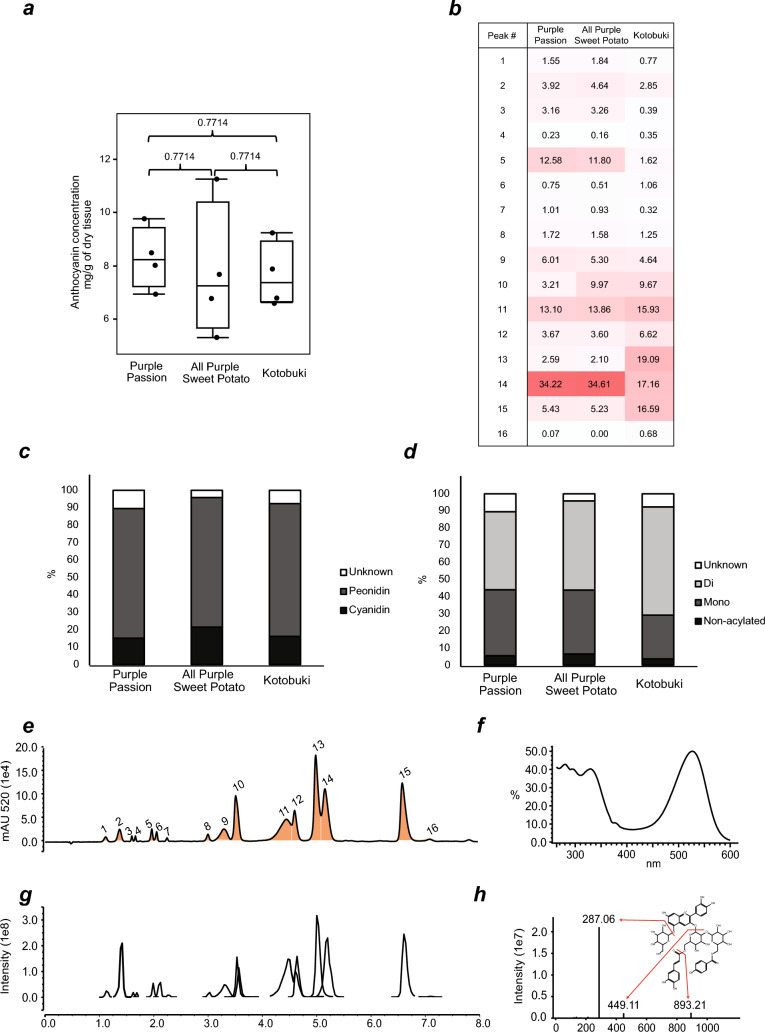
Anthocyanin diversity among the three assessed cultivars. (**a**) Box plot of total monomeric anthocyanin concentrations (cyanin equivalence) of sweet potato cultivars ‘Kotobuki’, ‘All Purple Sweet Potato’, and ‘Purple Passion’. KS test was utilized to assess p-values. (**b**) Heatmap showing percent of total anthocyanin content for each identified anthocyanin, based on UV–VIS 520 nm data. (**c**) Peonidin and cyanidin are the major aglycone classes found in the studied sweet potatoes (**d**) the majority of anthocyanins measured were acylated to some degree. (**e**–**h**) Representative LC-DAD-MS data from sweet potato cultivar ‘Kotobuki’ showing (**e**) a 520 nm UV–VIS chromatogram data is shown with (**f**) full absorption spectra of cyanidin 3-caffeoyl-p-hydroxybenzoyl sophoroside-5-glucoside and (**g**) an extracted ion chromatogram of anthocyanins identified across all sweet potato cultivars with (**h**) fragmentation pattern of cyanidin 3-caffeoyl-p-hydroxybenzoyl sophoroside-5-glucoside highlighting the cyanidin aglycone major fragment ion of *m/z* 287.06.

While relative anthocyanin concentration diversity among sweet potatoes is visually apparent, absolute concentrations are not. The three purple cultivars analyzed, ‘Kotobuki’ (7.37 median mg/g dry weight), ‘All Purple Sweet Potato’ (7.25 median mg/g dry weight), and ‘Purple Passion’ (8.23 median mg/g dry weight), contained levels of anthocyanins that were not significantly different from one another (Fig. 3a; Supplementary File 3), suggesting relative uniformity in the processes that lead to anthocyanin accumulation. A combination of spectrophotometric and mass spectrometric methods led to identification of 16 high-confidence anthocyanin peaks representing 18 anthocyanins (Table 1) (Fig. 3e–g). Sweet potatoes are known to produce acylated anthocyanins, which are chemically more stable to environmental changes than non-acylated anthocyanins. Acylation of anthocyanins can be determined using the ratio between the λ_max_ peak (~ 520 nm) and the acylation peak (~ 330 nm), as acylation results in increased absorptivity (hyperchromic effect) of the acylation peak^12^. An example of this phenomenon is shown for cyanidin 3-caffeoyl-p-hydroxybenzoyl sophoroside-5-glucoside (Fig. 3h). Sixteen of the 18 peaks were found to be acylated.

**Table 1:** Anthocyanins identified across the three purple sweet potato varieties. YGM stands for 'Yamagawamurasaki', the cultivar from which the previous sweet potato anthocyanins were elucidated via NMR. Retention time is abbreviated to RT. RT value was obtained as the average RT of the specific peak across all purple-fleshed varieties. Average mass error of the Orbitrap Q-Exactive instrument for MS1 anthocyanin masses was ~ 0.002 Da.

Peak no.	Tentative identification		Acylation class	RT (min)	Observed *m/z*			Supporting literature	NMR literature
					[M]+	Aglycone fragment ion	Qualifying fragment ions		
1	Cyanidin-3-sophoroside-5-glucoside	YGM 0A	Non	1.11	773.21	287.05	449.11, 611.16	^38^	^37^
2	Peonidin 3-sophoroside-5-glucoside	YGM 0B	Non	1.37	787.23	301.07	463.12, 625.17	^38^	
3	Cyanidin-3-p-hydroxybenzoyl sophoroside-5-glucoside		Mono	1.60	893.23	287.05	449.11, 731.18	^38^	
4	Cyanidin-3- caffeoyl sophoroside-5-glucoside	YGM 2	Mono	1.65	935.25	287.05	449.11, 773.19	^38^	^36^
5	Peonidin 3-p-hydroxybenzoyl sophoroside-5-glucoside		Mono	1.96	907.25	301.07	463.12, 745.20	^38^	
6	Peonidin 3-caffeoyl sophoroside-5-glucoside	YGM 5B	Mono	2.02	949.25	301.07	463.12, 787.21		^36^
7	Cyanidin-3-feruloyl sophoroside-5-glucoside		Mono	2.24	949.25	287.05	449.11, 787.21	^38^	
8	Peonidin-3-feruloyl sophoroside-5-glucoside		Mono	2.99	963.27	301.07	463.12, 801.22	^38^	
9	Cyanidin 3-caffeoyl sophoroside-5-glucoside	YGM 2	Mono	3.30	935.25	287.05	449.11, 773.19	^38^	^36^
10.1	Cyanidin 3-caffeoyl-p-hydroxybenzoyl sophoroside-5-glucoside	YGM 1A	Di	3.52	1055.27	287.06	449.11, 893.21	^38^	^37^
10.2	Cyanidin 3-dicaffeoyl sophoroside-5-glucoside	YGM 1B	Di	3.55	1097.28	287.06	449.10, 935.23		
11	Peonidin 3-caffeoyl sophoroside-5-glucoside	YGM 5B	Mono	4.45	949.25	301.07	463.12, 787.21	^38^	^36^
12.1	Peonidin 3-caffeoyl sophoroside-5-glucoside	YGM 5B	Mono	4.62	949.25	301.07	463.12, 787.21	^38^	^36^
12.2	Cyanidin-3-caffeoyl-feruloyl sophoroside-5-glucoside	YGM 3	Di	4.62	1111.29	287.05	449.11, 949.24	^38^	^37^
13	Peonidin 3-dicaffeoyl sophoroside-5-glucoside	YGM 4B	Di	5.01	1111.29	301.07	463.12, 949.24	^38^	^37^
14	Peonidin 3-caffeoyl-p-hydroxybenzoyl sophoroside-5-glucoside	YGM 5A	Di	5.15	1069.28	301.07	463.12, 907.23	^38^	^37^
15	Peonidin-3-caffeoyl-feruloyl sophoroside-5-glucoside	YGM 6	Di	6.62	1125.31	301.07	463.12, 963.25	^38^	^37^
16	Peonidin-3-caffeoyl-feruloyl sophoroside-5-glucoside *OR* Cyanidin 3-diferuloyl sophoroside-5-glucoside	YGM 6	Di	7.09	1125.31	287.05, 301.07	449.11, 963.25	^38^	^37^

Using multiple evidences—chemical formula, retention time, signature MS/MS fragments, anthocyanins detected in previous studies^36–38^—we structurally elucidated 17 of the 18 anthocyanins identified above (Table 1). These structures can be assigned at a confidence level of 2, defined as “probable structure by library spectrum match and/or by diagnostic evidence” as opposed to having an actual NMR structure (confidence level 1)^42^. The anthocyanidin core of ANT 16 could not be determined, as MS/MS peaks matching peonidin and cyanidin masses were present. Three acyl groups—feruloyl, *p*-hydroxybenzoyl, and caffeoyl—were observed. The high abundance anthocyanins were either peonidin or cyanidin derived and all were 3-sophoroside-5-glucosides with different levels of acylation. Through MS/MS fragmentation, low abundance petunidin, pelargonidin, and delphinidin derived anthocyanins were also detected. While these peaks were too low in abundance to be reliably portrayed in the UV–VIS data, specific signature fragments can still be utilized to tentatively identify low abundance anthocyanins.

### Anthocyanin content varies between the three purple-fleshed cultivars

The 18 identified anthocyanins make up > 90% of the anthocyanins within our samples across all cultivars sampled (Supplementary File 3). ‘Purple Passion’ and ‘All Purple Sweet Potato’ had very similar compositions, with only one anthocyanin (peak 6) being significantly different in its relative accumulation (two sample *t* test, p = 0.035) between the two samples (Fig. 3b; Supplementary File 3). In contrast, all but two of ‘Kotobuki’s’ identified anthocyanins (peaks 9, 10) were significantly different from anthocyanins in both ‘Purple Passion’ and ‘All Purple Sweet Potato’ (*t* test, p < 0.05) for both relative proportions and absolute peak areas (Supplementary File 3). Specific anthocyanins were further assessed for the aglycone type and acylation level. The aglycone type influences the visual pigmentation^43^ while acylation increases the stability of anthocyanins due to *pi* stacking interactions between the aromatic rings, resulting in intramolecular co-pigmentation^44,45^. The aglycone ratios of all three cultivars assessed were not significantly different, with peonidin making up the largest proportion of the anthocyanin types (Fig. 3c). Our results also show that ‘Kotobuki’ had higher diacylated anthocyanins (64% of the total anthocyanin peak area vs. 50% and 57%) and fewer monoacylated anthocyanins (26% vs. 40% and 34%) compared to ‘Purple Passion’ and ‘All Purple Sweet Potato’, respectively (Fig. 3d; Supplementary File 3). All three cultivars have similar levels of non-acylated anthocyanins (Fig. 3d).

### A deep learning approach identified hundreds of anthocyanin and flavonoid peaks in the untargeted metabolomics data

We next performed a more comprehensive assessment of flavonoid content across the different cultivars using the compound annotation tool CANOPUS^46^, which uses deep learning to classify MS/MS spectra into the hierarchical ChemOnt ontology^47^. As a hierarchical ontology, each classified compound is given a main, or “parent” class, as well as subsequent, more specific classifications (e.g. the class Flavonoids contains Flavonoid-3-*O*-glycosides, Flavones, Flavonols, etc. as subclasses). During development, CANOPUS’ deep learning algorithm was trained to identify characteristics of MS/MS spectra (fingerprints) among molecules of a structural class in the training datasets. For each experimental input spectra, CANOPUS generates multiple structural predictions, and each prediction is associated with a posterior probability score—a quantification of CANOPUS’ confidence in that prediction—that we used for filtering CANOPUS’ predictions (see “Methods”). CANOPUS is integrated into the Sirius software suite^39^, which is additionally capable of predicting compound formula and structure from MS/MS spectra.

We first validated the performance of CANOPUS on our data by having it classify the identified anthocyanins. Seventeen and 13 out of the 18 anthocyanins were correctly predicted to the “level 5” level (Flavonoid-*O*-glycosides), and the “most specific class” level (Anthocyanidin-3-*O*-glycosides), respectively (Supplementary Files 4, 5). Notably, two compounds received improbable formula identifications (e.g. formulas containing multiple nitrogens and chlorine), yet one of these compounds still received accurate structural annotation.

**Figure 4:**
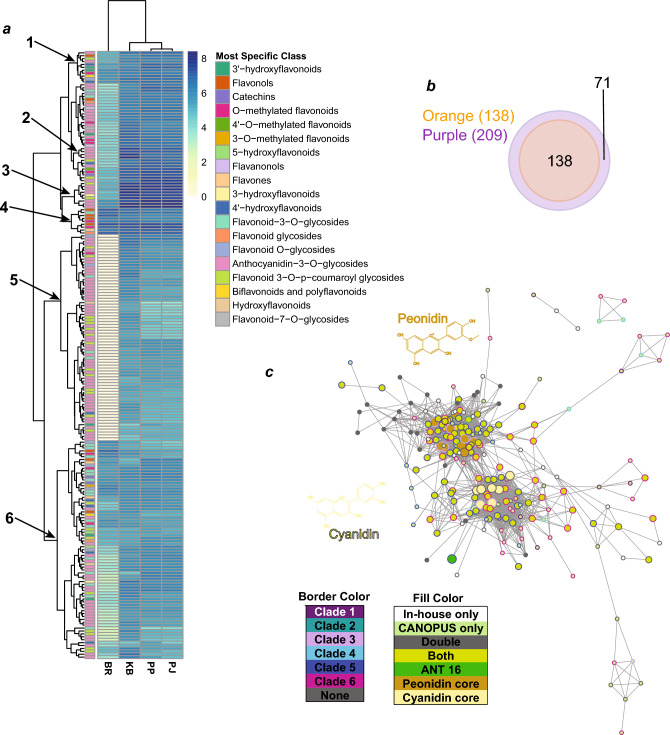
Flavonoid Abundance Across Cultivars and MS/MS Network: (**a**) Heatmap depicting the log10 average abundance of predicted flavonoids across sweet potato cultivars. *BR* Beauregard, *KB* Kotobuki, *PP* Purple Passion, *AP* All Purple Sweet Potato. Each flavonoid (row) is annotated with its most specific CANOPUS class, and clades of flavonoids with characteristic classifications and/or abundance patterns are numbered. (**b**) Venn Diagram depicting the presence/absence of predicted flavonoids across cultivars. The cultivar represented in the Orange set is Beauregard, and all other cultivars are represented in the Purple set. (**c**) MS/MS Molecular Network of CANOPUS-predicted and in house-predicted flavonoids. Node size and fill color represent flavonoid prediction methods. The largest nodes represent validated anthocyanins with a Cyanidin or Peonidin core (or ANT 16), medium-sized nodes (fill color of “Both”) were predicted as flavonoids by both methods, while small white nodes were predicted by in-house only, and small light green nodes were predicted by CANOPUS only. Gray nodes were doubly charged and thus not able to be predicted by CANOPUS. Border color represents grouping on the heatmap in (**a**).

Based on this analysis, we defined the lowest value of the CANOPUS posterior probability score for the above anthocyanins predicted as Flavonoids as the threshold for flavonoid classification. Of the 2172 singly charged peaks, CANOPUS predicted 238 flavonoids, 209 of which passed the posterior probability threshold. It is important to note that these numbers may be inflated due to metabolite processing considerations—such as the presence of adducts and the heuristic nature of peak detection—and the actual numbers of true, unique flavonoids may be lower. As expected, flavonoid peaks were present in larger numbers and at higher abundances in the purple-fleshed cultivars than in ‘Beauregard’ (Fig. 4a,b). The presence of 138 tentative flavonoid peaks in ‘Beauregard’ corroborates previous studies that have found low levels of flavonoids, anthocyanins, and flavonoid biosynthetic genes in orange-fleshed sweet potato cultivars^13^. Euclidean distance clustering of purple-fleshed cultivars based upon their flavonoid peak profiles revealed ‘Kotubuki’ to be the outlier, as ‘All Purple Sweet Potato’ and ‘Purple Passion’ had notably similar profiles. This result is in concordance with ‘Kotubuki’s’ significantly different anthocyanin levels when compared with ‘All Purple Sweet Potato’ and ‘Purple Passion’.

Euclidian distance clustering was also performed on the 209 flavonoids, based upon their abundance across cultivars (Fig. 4a). Mapping the most specific CANOPUS-generated structural annotation onto each flavonoid (rows of the heatmap) revealed regions of the heatmap with similar abundance patterns across cultivars, and either homogenous or heterogenous chemical annotations (Fig. 4a). Region 2, for example, exclusively contained anthocyanins, all with high levels in ‘Kotubuki’. Region 6, containing compounds absent in ‘Beauregard’, was dominated by anthocyanins, suggesting that, while ‘Beauregard’ does contain low levels of some anthocyanins, the majority of anthocyanin diversity seen in the purple cultivars is absent in ‘Beauregard’. This trend can be seen in additional regions of the network that contain compounds with low to no accumulation in ‘Beauregard’ (i.e. the bottom clade in region 6, and the area around region 4). Conversely, the regions of the network containing compounds abundant in ‘Beauregard’ (i.e. regions 1, 3, and the top clade in region 6) present a general lack of anthocyanins, in exchange for a diversity of other flavonoid chemistries, including 3′-hydroxyflavonoids (region 1), flavonols and O-methylated flavonoids (region 4), and flavonoid-3-*O*-glycosides (region 6). These results suggest that an expanded anthocyanin diversity is present in purple-fleshed sweet potato cultivars, and corroborate ‘Kotubuki’s maximum levels of certain anthocyanins among all purple-fleshed cultivars (region 2).

### Molecular networking of predicted flavonoids reveals the extent of flavonoid diversity

Anthocyanin glycosylations and acylations create signature fragments upon collision-induced dissociation in a mass spectrometer. Using the *m/z* ratios of pelargonidin, cyanidin, peonidin, petunidin, delphinidin and malvidin, and their corresponding glycosylated anthocyanin fragments as baits, we scanned the untargeted LC–MS/MS data from all cultivars for peaks that contained a core and a glycosylation fragment at relatively high intensities. This resulted in isolation of 274 peaks, comprising not just anthocyanins but likely also other flavonoids of similar fragmentation patterns (e.g. putative glycosides of quercitin, isorhamnetin, chrysoeriol, hesperitin)^13^ (e.g. Supplementary Fig. 2). Of these, 118 (43%) peaks were classified as Flavonoids by CANOPUS. These molecules included anthocyanins, as they are a subclass of Flavonoids. A total of 365 peaks—the union of fragment-based and CANOPUS-based predictions—were clustered using MS/MS molecular networking. Of these, 271 passed the 70% similarity cutoff to generate a network node, with all 18 identified anthocyanin peaks included. (Fig. 4c; Supplementary File 5). One flavonoid was removed due to an irregular after-decimal mass. This analysis clearly divided the anthocyanins into sub-networks based on individual aglycone fragments. The majority of flavonoids identified by both CANOPUS and in-house methods were highly connected to each other and to identified anthocyanins. In contrast to the dense network regions, the far-reaching areas of the network contained molecules predicted as uncommon subclasses of flavonoids, i.e. O-methylated flavonoids and flavonols. Additionally, the regions of the flavonoid heatmap (Fig. 4a) containing largely homogenous compound classifications (regions 2, 3, 5), had closely clustered nodes in the network, while regions with largely heterogenous classifications (1, 4, 6) had nodes dispersed across the network. The detection of metabolites with similar compound classifications, abundance patterns across cultivars, and MS/MS fragmentation patterns, is evidence that these methods may be uncovering various routes of flavonoid metabolism in sweet potatoes.

## Discussion

In recent years, better awareness of the nutritional properties of sweet potatoes has increased their popularity among the general public. While the carotenoid, vitamin and mineral content of orange-fleshed sweet potatoes has received more attention, anthocyanins and flavonoids from purple-fleshed varieties also have important health benefits of note^2,12^. Purple-fleshed sweet potatoes are not only attractive for general consumption but are also used in the health food, specialty chemicals, food processing, and cosmetics industries—the latter avenues also possibly fetching higher prices for the growers than traditional grocery markets. It is for these reasons that the cultivation of both orange and purple-fleshed sweet potatoes is being explored in the northern, cooler-climate regions of North America. In this study, we focused on characterizing the anthocyanin content of three purple-fleshed varieties to determine if substantial yield of desirable acylated anthocyanins can be obtained in northern latitudes.

We found ‘Kotobuki’ to have comparable total yield to the orange-fleshed standard ‘Beauregard’ (Fig. 1a), although this was attributed to the plants producing a many small-sized, “fingerling” roots, most of which were too small for retail grocery markets. Optimal growing conditions for the northern latitudes will need to be identified for growing such sweet potatoes directly for the consumer markets. In contrast, growing sweet potatoes for anthocyanin extraction is not fettered by their individual size. Our results suggest a yield of ~ 390 mg anthocyanins/100 g fresh weight of sweet potatoes and ~ 800 mg anthocyanins/100 g of lyophilized powder. These yields are at the medium–high end of the yields reported from other sweet potato varieties across different previous studies, respectively^14,38,48–51^, supporting a potential economic benefit to purple-fleshed sweet potato cultivation in northern latitudes.

We note some caveats in the anthocyanin estimates in this and possibly some previous studies. First, a recent study^34^ showed that the pH differential method underestimates the amount of monomeric anthocyanins, especially cinnamic acid conjugated derivatives, due to an alternate pathway of monomeric anthocyanin modification as the pH is increased in the pH differential method to 4.5. These alternatively modified anthocyanins are not distinguished from polymeric anthocyanins, thus leading to them not being estimated in the data. Second, this study also suggested that a commonly used protocol’s^33^ suggestion of 15-min incubation for equilibration of the external cyanin standard may lead to underestimation of anthocyanins. Further research is needed to determine the impact of these recent observations on overall anthocyanin estimation protocols. Finally, to mimic economical industrial extraction and to not increase the time and reagent cost by using additional solvent to diminishing yields, we did not perform re-extraction of anthocyanins or test the extraction efficiency under different growth, processing, extraction and estimation conditions—thus, our study specifically quantifies these molecules under the specified parameters. Nonetheless, these caveats would lead to under-estimation of the anthocyanin and flavonoid content, suggesting that the actual anthocyanin yields under exhausting conditions will be higher.

In addition to the anthocyanin yield, the types of anthocyanins produced are also important. Sixteen out of the 18 high-confidence anthocyanins we identified across the three purple cultivars were acylated, with most putative anthocyanin-like peaks having peonidin or cyanidin masses as the aglycones. There was no significant difference in anthocyanin concentrations, in ratios of cyanidin to peonidin, or acylated vs. non-acylated peak area ratios across the tested cultivars, indicating that the processes that lead to the accumulation of these compounds are similar. On the other hand, the balance between mono- and di-acylation was variable between the different cultivars. Of the three tested cultivars, ‘Kotobuki’, given its better growth characteristics, may be the best for acylated, especially di-acylated, anthocyanin extraction. Acylated anthocyanins find use in the food colorant industry, where color stability is important. Purple-fleshed sweet potatoes are also rich sources of flavonoids in general, as evidenced by inclusion of over three hundred LC–MS/MS peaks in the molecular network, fragmenting in consistence with flavonoid fragmentation patterns (Fig. 4c; Supplementary Fig. 2). This result substantially builds upon a previous study, which predicted 56 flavonoids (including 7 anthocyanins) from LC–MS data of different sweet potato cultivars^13^. The high interconnectivity among tentative flavonoids/anthocyanins, as well as the high degree of overlap between CANOPUS’ predicted flavonoids and those predicted in-house, suggests the power of computational methods as accurate structural classification tools for flavonoids.

In summary, our results suggest that purple-fleshed sweet potatoes grown in northern latitudes produce substantial levels of anthocyanins and flavonoids that can provide significant health benefit to consumers and economic benefit to the farmers if used for anthocyanin extraction. Formal studies comparing growth and nutritional characteristics of the same cultivars grown in warmer and cooler climates are needed. The slender build of the tested purple sweet potatoes may reduce their marketability in grocery stores, however, the high levels of acylated anthocyanins and flavonoids—coupled with their cultivation with reduced or no pesticides—make them attractive for other commercial applications. Further research would be needed to determine optimal cultivars and growing regimens suited for the northern soils and climates.

## Supplementary Information

Supplementary Figure 1.

Supplementary Figure 2.

Supplementary File 1.

Supplementary File 2.

Supplementary File 3.

Supplementary File 4.

Supplementary File 5.

## Data Availability

All source data used for data analysis in this manuscript are provided as Supplementary Files. The LC–MS files are uploaded to MetaboLights under the accession ID MTBLS2956.
