# Supplementary material for: Untargeted metabolomics of purple and orange-fleshed sweet potatoes reveals a large structural diversity of anthocyanins and flavonoids
Source: Sci Rep. 2021 Aug 12;11:16408. doi: 10.1038/s41598-021-95901-y (PMC8361111; doi:10.1038/s41598-021-95901-y)
Supplement: Supplementary file 1 — Supplementary Figure 1. [file 41598_2021_95901_MOESM1_ESM.pdf]

Supplementary Fig. 1

a

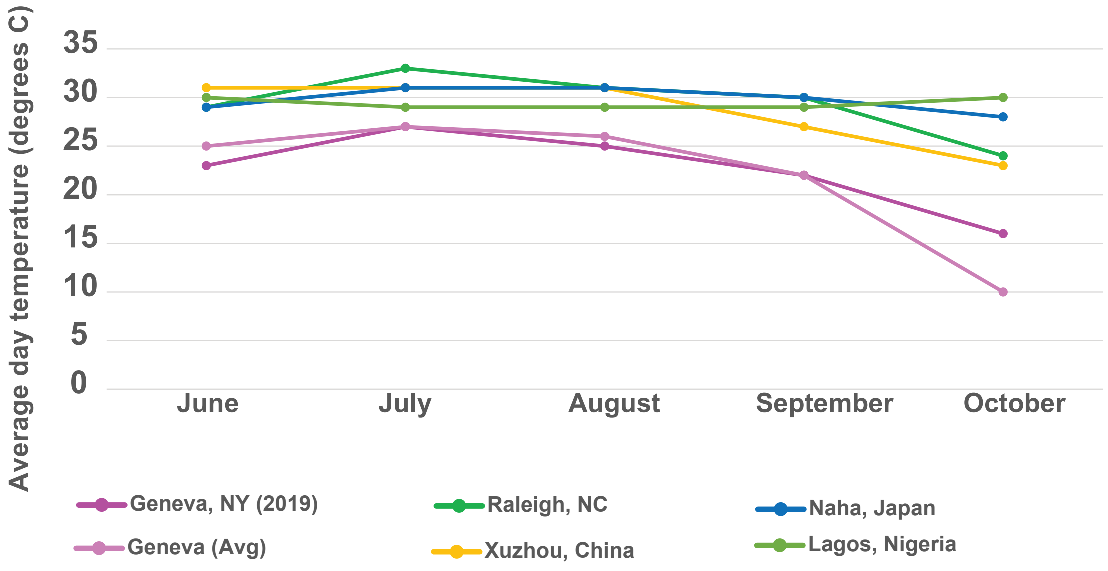

b

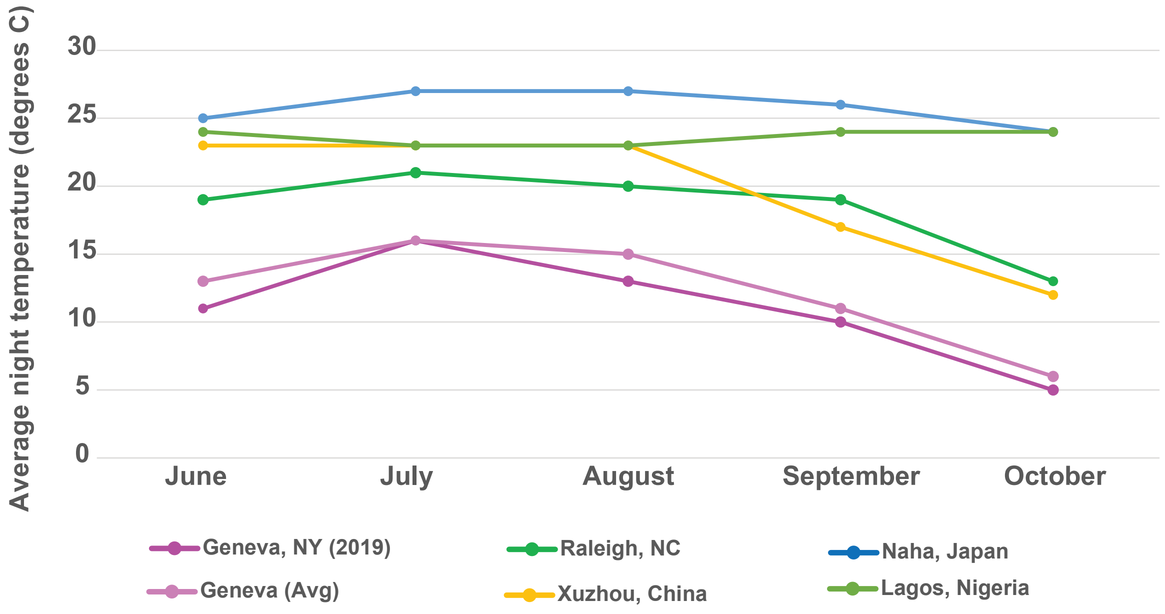

Supplementary Figure 1: Average high and low temperatures in different sweet potato cultivation areas. The average high (a) and low (b) temperatures at a given location are shown in lines of the same color. Data for Geneva (2019) was obtained from Weather Underground. All other data is high/low averages from 1985-2015 obtained from TimeAndDate.com.
