# Supplementary material for: Untargeted metabolomics of purple and orange-fleshed sweet potatoes reveals a large structural diversity of anthocyanins and flavonoids
Source: Sci Rep. 2021 Aug 12;11:16408. doi: 10.1038/s41598-021-95901-y (PMC8361111; doi:10.1038/s41598-021-95901-y)
Supplement: Supplementary file 2 — Supplementary Figure 2. [file 41598_2021_95901_MOESM2_ESM.pdf]

## *Supplementary Fig. 2*

**A.**

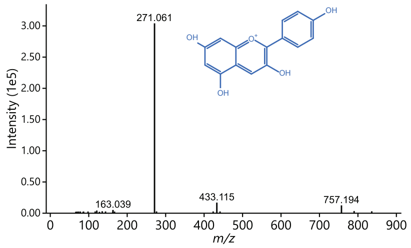


Pelargonidin 3-caffeoyl sophoroside 5-glucoside

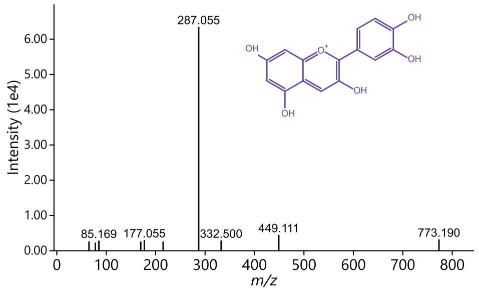


Cyanidin 3-caffeoyl sophoroside 5-glucoside

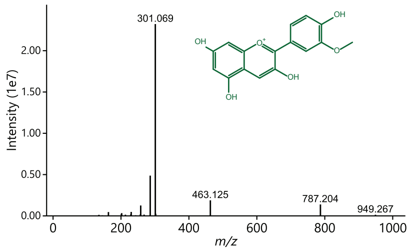


Peonidin 3-caffeoyl sophoroside 5-glucoside

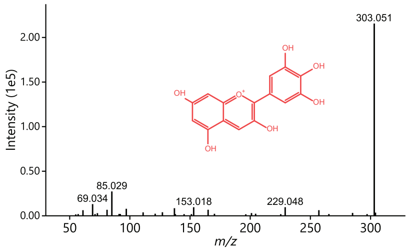


Delphinidin glucoside

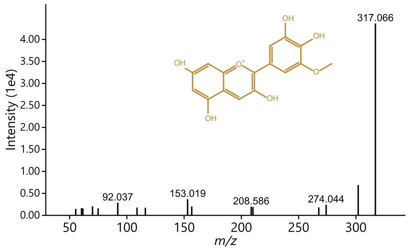


Petunidin glucoside

**B.**

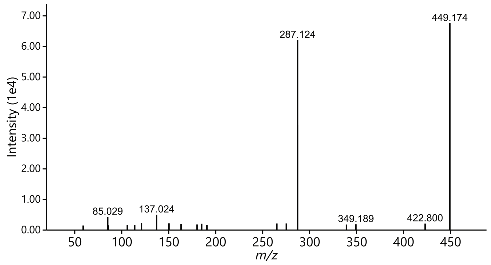


Kaempferol hexoside

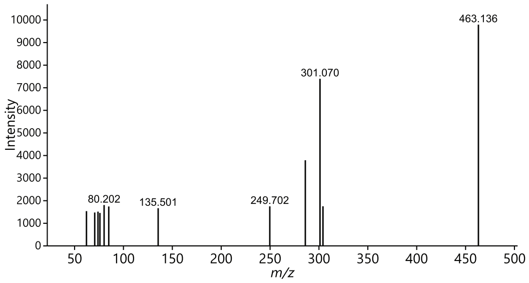


Chrysoeriol hexoside

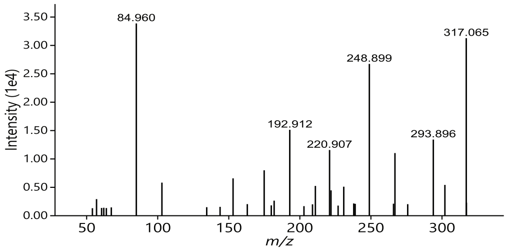


Isorhamnetin

**Supplementary Figure 2: Example MS/MS spectra** of (A) anthocyanins and (B) flavonoids of the same parent ion mass detected in our LC-MS datasets and present in the MS/MS molecular network. (A) Spectra of 3-caffeoyl sophoroside 5-glucoside of cyanidin, peonidin, pelargonidin, petunidin, delphidin. The aglycone originating from the flavylium core is the most intense fragment. (B) For the tentatively identified flavonoids, there are typically two intense fragments, one with the glucoside.
